# Comment on “Acupuncture Point “Hegu” (LI4) is Close to the Vascular Branch from the Superficial Branch of the Radial Nerve”

**DOI:** 10.1155/2021/9857079

**Published:** 2021-12-21

**Authors:** Kanae Umemoto, Munekazu Naito, Kaori Tano, Hayato Terayama, Taro Koike, Mika Ohmichi, Yusuke Ohmichi, Kou Sakabe, Takashi Nakano

**Affiliations:** ^1^Department of Anatomy, Aichi Medical University School of Medicine, 1-1 Yazakokarimata, Nagakute, Aichi, Japan; ^2^Department of Acupuncture and Moxibustion, Faculty of Health Science, Suzuka University of Medical Science, 1001-1 Kishioka, Suzuka, Mie, Japan; ^3^Department of Anatomy, Division of Basic Medical Science, Tokai University School of Medicine, 143 Shimokasuya, Isehara, Kanagawa, Japan; ^4^Department of Anatomy, Kansai Medical University, 2-5-1 Shinmachi, Hirakata, Osaka, Japan; ^5^Department of Anatomy II, Kanazawa Medical University, 1-1 Daigaku, Uchinada, Kahoku, Ishikawa, Japan

In the article titled “Acupuncture Point ‘Hegu' (LI4) is Close to the Vascular Branch from the Superficial Branch of the Radial Nerve” [1], the authors describe LI4 as being located at the base of the first and second metacarpal bones; however, the World Health Organization (WHO) describes LI4 as being on the dorsum of the hand radial to the midpoint of the second metacarpal bone [2] and the authors wish to make the following clarifications.

The contents and notations in textbooks on the topic of acupoints have been written separately in each country, and the standardization of meridian point locations was agreed in the Formal Consultation Meeting leading to the publication of the WHO Standard Acupuncture Point Locations in the Western Pacific Region by the WHO/WPRO in May 2008. The location of LI4 adopted during the meeting was different from that stated in textbooks in South Korea and Japan (i.e., at the base of the first and second metacarpal bones) [3]. In many historical books including “Huang Di Nei Jing Ling Shu (黄帝内経霊枢)”, which is one of the earliest written works on Traditional Chinese Medicine, the location of LI4 was stated to be “between the thumb and index finger.” It has been reported that the location of LI4 on “tai ping sheng hui fang (大平聖恵方)” (992) is considered to be the lower edge at the base of the first and second metacarpal bones [3, 4].
